# Localization and Expression of Nuclear Factor of Activated T-Cells 5 in Myoblasts Exposed to Pro-inflammatory Cytokines or Hyperosmolar Stress and in Biopsies from Myositis Patients

**DOI:** 10.3389/fphys.2018.00126

**Published:** 2018-02-21

**Authors:** Sandrine Herbelet, Elly De Vlieghere, Amanda Gonçalves, Boel De Paepe, Karsten Schmidt, Eline Nys, Laurens Weynants, Joachim Weis, Gert Van Peer, Jo Vandesompele, Jens Schmidt, Olivier De Wever, Jan L. De Bleecker

**Affiliations:** ^1^Department of Neurology, Ghent University and Ghent University Hospital, Ghent, Belgium; ^2^Cancer Research Institute Ghent and Department of Radiation Oncology and Experimental Cancer Research, Ghent University, Ghent, Belgium; ^3^VIB Inflammation Research Center, Ghent, Belgium; ^4^Department of Biomedical Molecular Biology, Ghent University, Ghent, Belgium; ^5^VIB Bio Imaging Core Gent, Ghent, Belgium; ^6^Department of Neurology and Department of Experimental and Clinical Neuroimmunology, University of Göttingen, Göttingen, Germany; ^7^Institute of Neuropathology, RWTH Aachen Medical School, Aachen, Germany; ^8^Center for Medical Genetics and Cancer Research Institute Ghent, Ghent, Belgium

**Keywords:** NFAT5, myoblasts, hyperosmolar stress, pro-inflammatory cytokines, myositis

## Abstract

**Aims:** Regeneration in skeletal muscle relies on regulated myoblast migration and differentiation, in which the transcription factor nuclear factor of activated T-cells 5 (NFAT5) participates. Impaired muscle regeneration and chronic inflammation are prevalent in myositis. Little is known about the impact of inflammation on NFAT5 localization and expression in this group of diseases. The goal of this study was to investigate NFAT5 physiology in unaffected myoblasts exposed to cytokine or hyperosmolar stress and in myositis.

**Methods:** NFAT5 intracellular localization and expression were studied *in vitro* using a cell culture model of myositis. Myoblasts were exposed to DMEM solutions enriched with pro-inflammatory cytokines IFN-γ with IL-1β or hyperosmolar DMEM obtained by NaCl supplementation. NFAT5 localization was visualized using immunohistochemistry (IHC) and Western blotting (WB) in fractionated cell lysates. NFAT5 expression was assessed by WB and RT-qPCR. *In vivo* localization and expression of NFAT5 were studied in muscle biopsies of patients diagnosed with polymyositis (*n* = 6), dermatomyositis (*n* = 10), inclusion body myositis (*n* = 11) and were compared to NFAT5 localization and expression in non-myopathic controls (*n* = 13). Muscle biopsies were studied by means of quantitative IHC and WB of total protein extracts.

**Results:** In unaffected myoblasts, hyperosmolar stress ensues in NFAT5 nuclear translocation and increased NFAT5 mRNA and protein expression. In contrast, pro-inflammatory cytokines did not lead to NFAT5 nuclear translocation nor increased expression. Cytokines IL-1β with IFN-γ induced colocalization of NFAT5 with histone deacetylase 6 (HDAC6), involved in cell motility. In muscle biopsies from dermatomyositis and polymyositis patients, NFAT5 colocalized with HDAC6, while in IBM, this was often absent.

**Conclusions:** Our data suggest impaired NFAT5 localization and expression in unaffected myoblasts in response to inflammation. This disturbed myogenic NFAT5 physiology could possibly explain deleterious effects on muscle regeneration in myositis.

## Introduction

Idiopathic inflammatory myopathies (IIM) are auto-immune muscle diseases comprising polymyositis (PM), dermatomyositis (DM), inclusion body myositis (IBM), overlap myositis (OM), and immune mediated necrotizing myopathy (IMNM) (Dalakas, 1991; Dalakas and Hohlfeld, 2003; Hoogendijk et al., 2004; De Bleecker et al., 2013). These diseases are characterized by chronic inflammation and presence of pro-inflammatory cytokines. DM is a complement mediated endotheliopathy whereas IBM and PM are cytotoxic T-cell mediated diseases (De Bleecker and Engel, 1995). In IBM, protein misfolding and autophagosome dysfunction do occur (Benveniste et al., 2015). In IIM, affected muscle fibers suffer necrosis (Loell and Lundberg, 2011). Attempts to regenerate muscle tissue in IIM are compromised. Impaired proliferation and altered fusion capacity has been described in primary cell cultures from patients diagnosed with IIM (Cseri et al., 2015).

Skeletal muscle tissue regeneration occurs in five phases: degeneration by necrosis, transient inflammation, regeneration, remodeling, and functional repair (Musarò, 2014). After activation, muscle stem cells become myoblasts, which will fuse with damaged myofibers or with each other, forming new myofibers (Hawke and Garry, 2001; Musarò, 2014). Transient promyogenic inflammation is tightly regulated and perturbation in the magnitude of the pro-inflammatory cytokines induces chronic inflammation, which is deleterious to muscle repair. One of the possible mechanisms explored in the past to explain this effect is the activation of nuclear factor-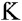
B (NF-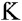
B) by pro-inflammatory cytokines, interfering with the expression of muscle proteins in differentiating myoblasts (Langen et al., 2001).

Nuclear factor of activated T-cells 5 (NFAT5) belongs to the Rel family of transcription factors and is closely related to NF-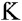
B (López-Rodríguez et al., 2001). It is expressed in skeletal muscle, kidney cells, lymphocytes, thymus and testes (Miyakawa et al., 1998; López-Rodríguez et al., 1999; Trama et al., 2000). Five different isotypes have been described for NFAT5: NFAT5a, NFAT5b, NFAT5c, NFAT5d1, and NFAT5d2 (Eisenhaber et al., 2011). Approximately half of endogenous NFAT5 under isotonic conditions is isoform NFAT5a, attached to the plasma membrane by palmitoylation and myristoylation. NFAT5b and NFAT5c are diffusely present in the cytoplasm. In NFAT5a, only the plasma-membrane bound fraction is sensitive to osmolar changes, diffusing to the nucleus upon stimulation (Eisenhaber et al., 2011). NFAT5 in general is tonicity sensitive in skeletal muscle cells and is involved in myoblast migration and differentiation (O'Connor et al., 2007). Cell motility and migration is regulated by histone deacetylase 6 (HDAC6). Besides, NFAT5 regulates heat-shock protein 90 (Hsp90), involved in IIM (Boyault et al., 2007; Zhang et al., 2007; De Paepe et al., 2012).

In virtually all cells, maintaining cellular homeostasis is met with nuclear translocation of NFAT5 and production of organic osmolytes (Evans et al., 2009), restoring homeostasis without disturbing cell function. The NFAT5 pathway can be activated by osmotic shock or receptor activation, yielding a different gene activation program (Kim N. H. et al., 2014). Extracellular hypertonicity drains intracellular water to the extracellular medium causing cell shrinkage (Lang et al., 1998) and DNA damage. This is counteracted by electrolyte uptake (i.e., regulatory volume increase) leading to high intracellular ionic strength (Lang et al., 1998; Alfieri and Petronini, 2007). The latter is neutralized by uptake and production of osmolytes such as sorbitol, betaine and taurine (Brown, 1976, 1978; Lang et al., 1998) after NFAT5 translocation to the nucleus (Ko et al., 2000; Dahl et al., 2001). NFAT5 attempts to rescue osmolarity by restoring cell volume and protecting DNA of incoming K^+^ and Na^+^ ions (Ferraris et al., 1996; Miyakawa et al., 1998). NFAT5 is an activator of tumor necrosis factor (TNF), lymphotoxin β (LTβ) and heat shock protein 70 (HSP70) transcription (Woo et al., 2002; Esensten et al., 2005; Creus et al., 2012). Both TNF-α, LTβ and HSP70 are involved in IIM (De Bleecker et al., 1999; Creus et al., 2012; De Paepe et al., 2012).

In this study we have hypothesized that there is impaired NFAT5 localization and expression in myoblasts exposed to pro-inflammatory cytokines IFN-γ with IL-1β since IIM are characterized by chronic inflammation, deleterious to muscle regeneration. In myoblasts exposed to hyperosmolar conditions, we expect NFAT5 translocation to the nucleus and increased NFAT5 protein expression. According to our hypothesis, we expect impaired NFAT5 localization and expression in muscle biopsies from patients diagnosed with IIM. Exploring the putative colocalization of NFAT5 with HDAC6, involved in cell motility, in both myoblasts and muscle biopsies may further our understanding of NFAT5's cellular interactions in skeletal muscle tissue under pro-inflammatory or hyperosmolar conditions.

## Materials and methods

### *In vitro* culture of myoblasts from non-myopathic individuals

Two primary myoblast cell cultures obtained from the Myobank Banque D'ADN, France, were used for *in vitro* experimentation and were kept under passage 12, to avoid cellular senescence. Myoblasts obtained from unaffected individuals were labeled “UMyo.” Hence, cell cultures UMyo1 and UMyo2 originated from unaffected individuals (Supplementary Table 1). Consent was obtained from all subjects and the study was approved by local Ethic Committees. All cultures were grown in DMEM containing glucose and 1% L-glutamine (Life technologies, Carlsbad, USA), supplemented with 10% FCS (Cambrex, Bioscience, Walkersville, USA), penicillin (50 IU/ml) + streptomycin (50 mg/ml) (Gibco, Invitrogen, Carlsbad, USA) (“DMEM”). Myogenicity was assessed in UMyo by IHC using an antibody against CD56 (neural cell adhesion molecule, NCAM) (Supplementary Table 2 and Supplementary Figure 1A, UMyo1).

UMyo7 and UMyo8 were obtained from the Laboratory of Experimental and Clinical Neuroimmunology in Göttingen, Germany. These cultures were grown in Skeletal Muscle Cell Growth Medium + Supplement Mix (Promocell, Heidelberg, Germany) at 37°C and 5% CO_2_.

### Exposure of unaffected myoblasts to pro-inflammatory cytokines IFN-γ with IL-1β or hyperosmolar NaCl concentrations

UMyo1 and UMyo2 were transferred at 80% confluence to 8-chamber slides for IHC studies and to 175-cm^2^ flasks with DMEM for WB and RT-qPCR. Two 175-cm^2^ flasks were used per condition to obtain sufficient amounts of cells for protein and mRNA extraction. To mimic inflammatory conditions seen in myositis, myoblasts were exposed to a mixture of pro-inflammatory cytokines IFN-γ with IL-1β diluted in DMEM to respective concentrations of 300 U/ml and 20 ng/ml (R&D Systems, Minneapolis, USA) for 7 or 24 h (Schmidt et al., 2008). Unstimulated myoblasts served as controls.

To examine the impact of differentiation on NFAT5 physiology in myoblasts, UMyo7 and UMyo8 were transferred to 8-chamber slides (LabTek II, Nunc, Penfield, USA) with DMEM supplemented with 0.5% chick embryo extract (CEE, Accurate, Westbury, USA). At 80% confluence, fusion was induced by adding heat-treated horse serum for 48 h.

In DMEM, osmolarity was 110 mM (280 mOsm/L) with a NaCl concentration of 6.40 mg/ml. Preparing hyperosmolar DMEM solutions was performed by adding 1.10 mg/ml NaCl, to obtain an increase of 18 mM, raising the concentration of the solution to 128 mM (333 mOsm/L). This solution is referred to as “DMEM18” and corresponds to the change in osmolarity after addition of cytokines to DMEM. Addition of 3.50 mg/ml NaCl to DMEM to obtain an increase of 60 mM of the solution, resulted in a final osmolarity of 443 mOsm/L. The increase in 60 mM was based on Na^+^ concentrations in blood in the range of 135–145 mM combined with the recent insight pointing to excess salt intake as a worsening factor in autoimmune diseases (Sigaux et al., 2017). This solution is designated as “DMEM60.” DMEM supplemented with cytokine mixture IFN-γ with IL-1β displayed an osmolarity of 339 mOsm/L and was labeled as “DMEMCyto.” To study the impact of cytokines only, addition of DMEM18 (333 mOsm/L) to untreated cells yielded hyperosmolar controls. This hyperosmolar condition was used as control to compensate for the osmolar change induced in DMEMCyto. Osmolarity of the media supplemented with NaCl or cytokines was measured using an osmometer (Osmometer Automatic, Knauer, RS45105-0, Berlin, Germany).

### Patients

Diagnostic limb muscle biopsies (*n* = 27) were performed after written informed consent had been obtained from patients or their legal representatives with PM (*n* = 6), DM (*n* = 10), IBM (*n* = 11) from the University Hospitals of Ghent and Antwerp (Belgium) and the Institute of Neuropathology, RWTH Aachen Medical School (Germany). All patients fulfilled the conventional criteria for diagnosis (Dalakas and Hohlfeld, 2003; De Bleecker et al., 2013). From the cryoblocks remaining after diagnostic work-up, 6 μm transversal sections were obtained for IHC and protein extraction. PM is only diagnosed when non-necrotic invaded muscle fibers are present in the diagnostic biopsy and patients have reacted to immunotherapy. At the time of biopsy, all patients were free of immunotherapy and all of them had a progressive disease. The patients' medical files were searched for concomitant diseases (Supplementary Table 3). As NFAT5 is elevated in diabetic patients (Yang et al., 2006), this has to be taken into account during the analysis of the results. The same cautious approach is required if other autoimmune diseases are present. Muscle biopsies from healthy individuals with no clinical, electromyographic, or histologic evidence of myopathy and free from any known other disease served as controls (*n* = 13). The study was approved by the Ghent University Hospital Medical Ethics Committee and the RWTH Aachen Medical School Ethics Committee.

### Muscle biopsies, quantitative immunofluorescent double and triple staining, confocal microscopy, and image analysis

Frozen 6 μm transversal muscle tissue sections were used for double immunofluorescence (IF) performed as described previously (De Bleecker et al., 2002). Supplementary Table 2 summarizes antibodies and concentrations used during staining for 1 h. Secondary staining was performed for 1 h with secondary antibodies linked to the fluorescence markers AlexaFluor-488 (green), FITC (green), AlexaFluor-555 (red) (Invitrogen, Waltham, USA) or Cy3 (red) (Jackson ImmunoResearch Laboratories West Grove, USA). Sections were imaged with a Leica SP8 AOBS confocal microscope (Leica, Mannheim, Germany). Images were taken using a 63X HCX PL Apo 1.4 NA oil objective. Z-sections were made at the resolution limit, in this case 126 nm, to produce a high-resolution stack suitable for colocalization measurements. Images were acquired in a sequential mode, scanned as 246 X 246 nm per pixel. To test for antibody specificity, NFAT5 siRNA studies were completed. As NFAT5 Rabbit yielded unspecific binding in myonuclei, NFAT5 Goat was selected as the antibody of choice (Supplementary Figure 1B). NFAT5 Goat binds to all five NFAT5 isoforms. Amino acid sequence analysis by BLAST (Basic Local Alignment Search Tool, NIH, Bethesda, USA) of the NFAT5 Rabbit antibody yielded recognition of two proteins, rendering the antibody aspecific to NFAT5 (LLVSLQNQGN NLTGSF). The NFAT5 Goat was specific to NFAT5 (MPSDFISLLS ADLDLESPKS LYSRESVYDL LPKELQLPPS RETSVASMSQ).

### Quantitative western blotting (WB)

Total protein extracts were prepared from 175-cm2 flasks using appropriate lysis buffer (Laemmli 1x; 2-Mercaptoethanol 0.1%, Bromophenol Blue 0.01%, Glycerol 10%, SDS 2%, Tris-HCl 60 mM pH 6.8) followed by sonification. For patients' biopsies, protein extracts were prepared by homogenizing frozen muscle samples in 2 volumes of extraction buffer (50 mM TrisHCl, 2 mM EDTA pH 7.4) supplemented with protease inhibitor (TM mini protease inhibitor cocktail; Roche, Bazel, Switzerland). To pellet debris, samples were centrifuged at 2,000 g for 10 min. Proteins were transferred to nitrocellulose membranes by electroblotting and incubated with primary antibodies (Hendrix et al., 2010) (Supplementary Table 4) and anti-GADPH (Abcam, Cambridge, Massachusetts, USA) to correct for protein concentration between samples. PARP was used as a nuclear control in WB of fractionated cell lysates (BD Biosciences, Franklin Lakes, New Jersey, USA) and GAPDH as a cytosolic marker. Immunoreaction was visualized using chemiluminescence (WesternBright™ Sirius, Advansta, Menlo Park, California, USA) and Proxima 2650 (Isogen Life Science, De Meern, The Netherlands).

In cell cultures, cell fractionation into nuclear and cytosolic parts was performed by adding a hypotonic buffer (20 mM HEPES, 20% glycerol, 10 mM NaCl, 1.5 mM MgCl_2_, 0.2 mM EDTA, 0.1% Triton x-100 and MQ) for cell lysis. Nuclei were separated into a pellet after gentle centrifugation at 800 rpm for 10′. After resuspending the pellet in a hypertonic buffer (cf. hypotonic buffer with 500 mM NaCl instead of 10 mM NaCl), rotating it and spinning down, a supernatant containing the nuclear fraction was obtained. NFAT5 antibody selectivity for WB was assessed using siRNA NFAT5 (h): sc-43968 (Santa Cruz Biotechnology, Santa Cruz, California, USA) in one cell culture at 20 nM starting from a stock solution at 10 μM. Scrambled RNA (scRNA) was used as internal control for siRNA specificity. ScRNA was prepared by diluting 2 μL in 2 mL serum free DMEM following the supplier's protocol. Lipofectamine 2000 (Invitrogen) was used for transfection by diluting 75 mL in 2 mL serum free DMEM followed by incubation for 5′ at RT. As lipofectamine was produced in duplicate, each vial was added to the respective siRNA and scRNA solutions and gently mixed for 20′ at RT. In one 75 cm^2^ flask with confluent layer, the siRNA with lipofectamine was added. In the second 75 cm^2^ flask with confluent layer, scRNA with lipofectamine was added. Both flasks were incubated for 24 h at 37°C with 5% CO_2_. We used a DMEM solution with an osmolarity increased to 135 mM and results were obtained by WB of total cell extractions.

### RT-qPCR

One 175-cm^2^ flask per treatment condition was grown to full confluency and cell cultures were exposed to cytokines or hyperosmolar stress for 7 or 24 h. Total lysis of cell cultures was obtained by following a well-described protocol (Vandesompele et al., 2002) with a minimum purity of 1.9/2 measured by the A260/A280 ratio on BioDrop™ Touch Duo PC (Harvard Bioscience Inc., Holliston, Massachusetts, USA). Results in line with the MIQE guidelines (Bustin et al., 2009) were considered relevant after calculation with qBase+ Software version 2.6 (www.qbaseplus.com) (Biogazelle, Zwijnaarde, Belgium) (Hellemans et al., 2007). geNorm was used to determine the most stable reference genes from a set of tested candidate reference genes (Supplementary Table 5).

### Statistical analysis

For statistical analysis of quantitative confocal microscopy images, the Mann Whitney U test was performed in SPSS 23.0 (IBM, Armonk, New York, USA), *p* < 0.05 were considered statistically significant. The statistical analysis of colocalization in confocal images was performed with Volocity 6.1.3 (Perkin Elmer, Coventry, UK). Background levels were obtained by measuring the voxel count of the signal outside the cells and subtracted. Colocalization indexes M1 (NFAT5) and M2 (HDAC6) (which indicate the quantity of colocalization in each individual channel), Mander's Overlap coefficient (R) and Pearson's correlation coefficient (*r*) were calculated using the appropriate algorithm in the Volocity software package. Pearson's correlation was solely chosen as a thresholding method because measurements above background in both channels are included in the calculations (Costes et al., 2004). M1 stands for the quantity of red signal in the green channel, and M2 for the quantity of green signal in the red channel. One-way ANOVA with Tukey's multiple comparison test was applied to the values obtained from RT-qPCR analyzed by qBase+.

## Results

In UMyo, myoblast myogenicity was investigated by IHC. Double IF staining with NFAT5 and NCAM yielded superposition of both dyes after merging the immunofluorescent detection channels (Supplementary Figure 1A).

All results described below were obtained in cell cultures UMyo1 and UMyo2 where differentiation occurred by spontaneous fusion, without any differentiation medium (Supplementary Figure 2). In both cell cultures, myoblasts with single myonuclei could be observed as well as nascent myotubes harboring different myonuclei per cell, albeit to a lesser extent.

### NFAT5 localization in unaffected hyperosmolar stressed myoblasts

**Figure 1:**
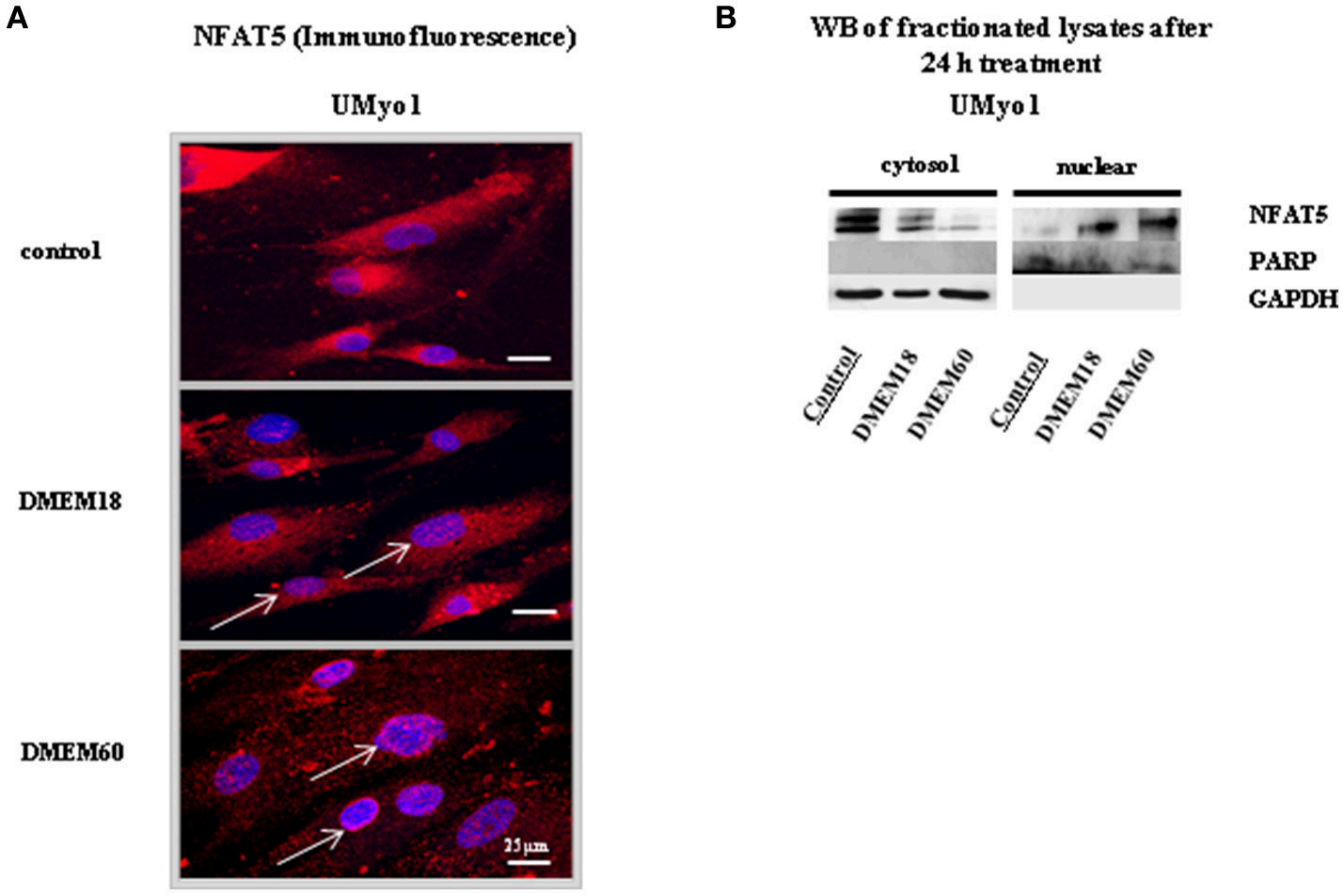
NFAT5 localization in unaffected myoblasts after 24 h exposure to hyperosmolar conditions. **(A)** Immunofluorescent staining was performed in UMyo1 (unaffected myoblasts) with NFAT5 Goat antibody, followed by a Cy3 (red)-labeled secondary antibody. Myonuclei were counterstained with DAPI staining (blue). White arrows point at increased NFAT5 nuclear staining in UMyo1 exposed to DMEM18 or DMEM60 (DMEM with a total increase in osmolarity of 18 or 60 mM) (*n* = 3). **(B)** By WB, NFAT5 immunoreactivity increased in the nuclear fraction of UMyo1 after hyperosmolar stress. Cytosolic NFAT5 immunoreactivity decreased with increasing hyperosmolar stress (*n* = 1).

The impact of osmolar stress on NFAT5 localization was studied in cultured myoblasts. Two primary myoblast cultures from non-myopathic individuals (UMyo) were exposed to hyperosmolar DMEM. NFAT5 localization was studied by IHC and WB of fractionated cell lysates. In WB, PARP was used as a marker for the nuclear fraction, GAPDH as a cytosolic marker. Results are displayed in Figure 1. Variation in results amongst different cell cultures are displayed in Supplementary Figure 3.

By IHC and WB in UMyo1, NFAT5 was merely localized in the cytoplasm of untreated myoblasts (*n* = 3). Exposure to increasing hyperosmolar conditions showed a decrease in NFAT5 cytoplasmic staining (*n* = 3) and NFAT5 immunoreactivity in the cytosolic fraction (*n* = 1). NFAT5 nuclear staining increased (*n* = 3) along with NFAT5 immunoreactivity in the nuclear fraction (*n* = 1). In WB specifically, PARP was merely expressed in the nuclear fraction and GAPDH immunoreactivity was mainly visible in the cytosolic compartment (Figure 1).

Variation in NFAT5 localization in UMyo was explored by IHC after exposure to DMEM60. In all UMyo cultures, NFAT5 cytoplasmic staining appeared in a punctate pattern in untreated conditions. NFAT5 nuclear staining was observed in all UMyo after hyperosmolar stress (Supplementary Figure 3A). In UMyo7 and UMyo8, where DMEM was supplemented with heat-treated horse serum, addition of heat-treated horse serum did not influence NFAT5 localization as visualized in UMyo8 exposed to DMEM18 or DMEM60 (Supplementary Figure 1C).

### Impaired NFAT5 nuclear localization in unaffected myoblasts exposed to pro-inflammatory cytokines IFN-γ with IL-1β

**Figure 2:**
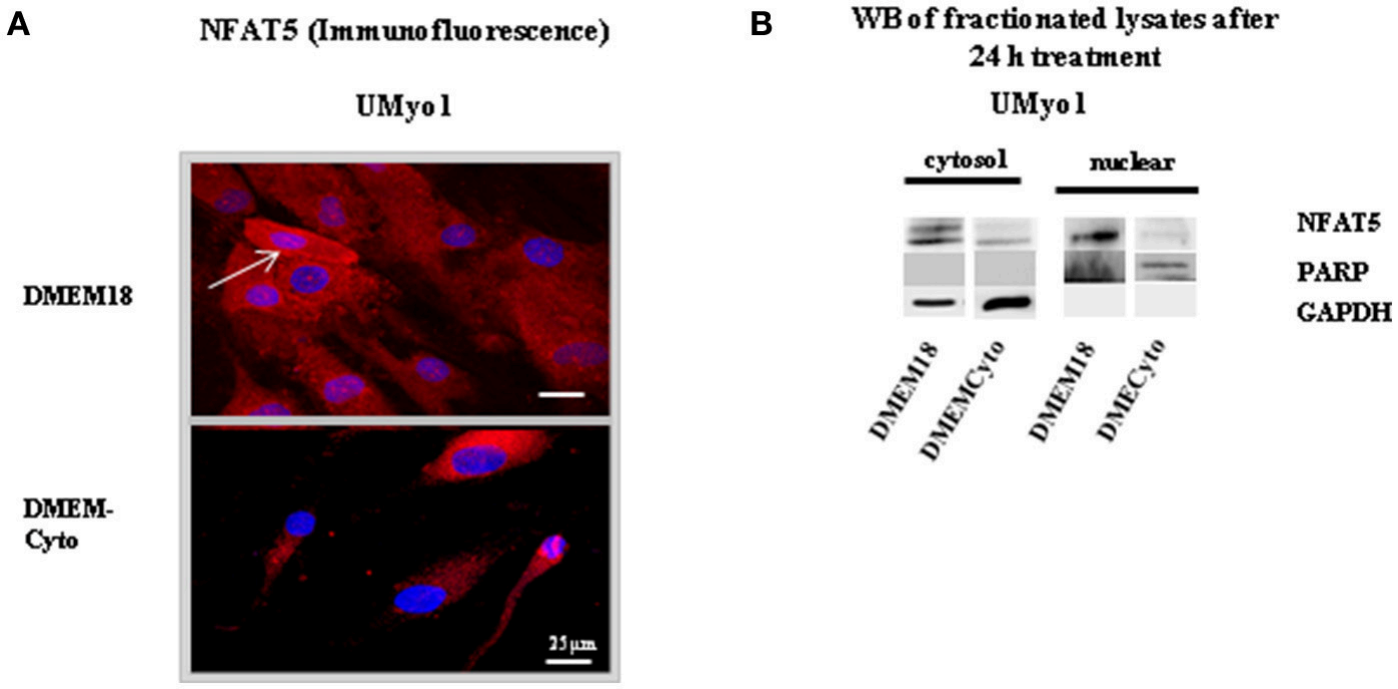
NFAT5 localization in unaffected myoblasts after 24 h exposure to DMEM supplemented with pro-inflammatory cytokines IFN-γ with IL-1β. **(A)** Immunofluorescent staining was performed in UMyo1 (unaffected myoblasts) with NFAT5 Goat antibody, followed by a Cy3 (red)-labeled secondary antibody. Myonuclei were counterstained with DAPI staining (blue). NFAT5 nuclear staining was often absent in UMyo1 exposed to DMEMCyto (DMEM enriched with IFN-γ with IL-1β). The white arrow points at increased NFAT5 nuclear staining in UMyo1 exposed to DMEM18 (DMEM with a total increase in osmolarity of 18 mM) (*n* = 3). **(B)** By WB, NFAT5 immunoreactivity decreased in the cytosolic and nuclear fraction of UMyo1 (n = 1).

The influence of cytokine stress on NFAT5 localization was studied in cultured myoblasts. Two primary myoblast cultures from non-myopathic individuals were exposed to DMEM enriched with pro-inflammatory cytokine mixture IFN-γ (300 U/ml) with IL-1β (20 ng/ml) (DMEMCyto). NFAT5 localization was studied by IHC and WB of fractionated cell lysates. In WB, PARP was used as a marker for the nuclear fraction, GAPDH as a cytosolic marker. To study the impact of cytokines alone, addition of DMEM18 to untreated cells yielded hyperosmolar controls. This hyperosmolar condition was used as control to compensate for the osmolar change induced in DMEMCyto. Results are displayed in Figure 2.

By IHC and WB in UMyo1, control myoblasts showed NFAT5 cytoplasmic and nuclear staining and NFAT5 immunoreactivity in the cytosolic and nuclear compartments (cf. 3.1). In UMyo1 exposed to pro-inflammatory cytokines IFN-γ with IL-1β, NFAT5 nuclear staining (*n* = 3) and immunoreactivity (*n* = 1) were strongly decreased in almost all cells (Figure 2).

### NFAT5 expression in unaffected myoblasts exposed to hyperosmolar or cytokine stress

**Figure 3:**
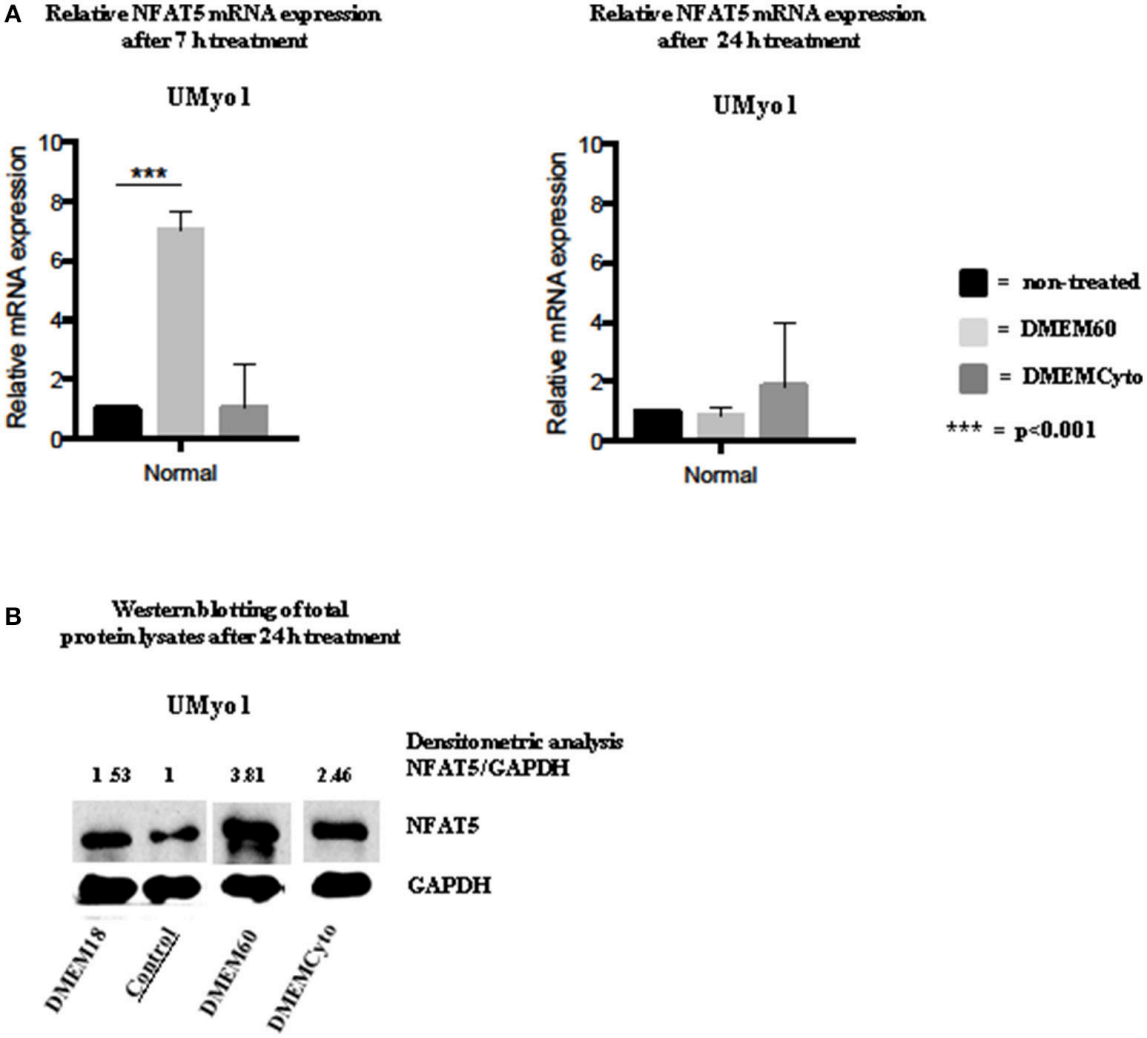
NFAT5 mRNA and protein expression in unaffected myoblasts exposed to hyperosmolar or pro-inflammatory conditions. **(A)** In UMyo1 (unaffected myoblasts), exposure to DMEM60 for 7 h significantly increased NFAT5 mRNA expression (*p* < 0.001). In contrast, NFAT5 mRNA was not increased in UMyo1 after 24 h hyperosmolar treatment and after 7 or 24 h exposure to DMEMCyto (*n* = 3). Untreated UMyo1 were used as control. **(B)** By WB of total cell lysates, an increase in NFAT5 protein levels was measured in UMyo1 after 24 h hyperosmolar challenge (*n* = 3).

NFAT5 expression was studied by means of WB and RT-qPCR. Firstly, RT-qPCR explored the presence of NFAT5 mRNA after 7 and 24 h of exposure to cytokine or hyperosmolar stress. Secondly, WB was performed on total cell lysates exposed to hyperosmolar or cytokine stress for 24 h, to study protein expression. The results are presented in Figure 3 and Supplementary Figure 3B.

In UMyo1, a fold change in NFAT5 mRNA expression of 7.04 (±1.53) (*p* < 0.001) was measured for ^*NFAT*5^ after 7 h of exposure to DMEM60, with subsequent significant increase in NFAT5 protein level (*n* = 3). Following conditions yielded no significant increase in NFAT5 mRNA expression: exposure for 24 h to DMEM60, 7 or 24 h exposure to DMEMCyto. Exposure for 24 h to DMEMCyto had a minor influence on NFAT5 protein expression (*n* = 3) (Figure 3).

Variation in NFAT5 protein expression amongst cell cultures was studied by WB of total cell lysates of UMyo1 and UMyo2. DMEM60 increased significantly NFAT5 protein expression (*n* = 3) (Supplementary Figure 3B).

### NFAT5 colocalization with HDAC6 in unaffected myoblasts exposed to pro-inflammatory cytokines IFN-γ with IL-1β

**Figure 4:**
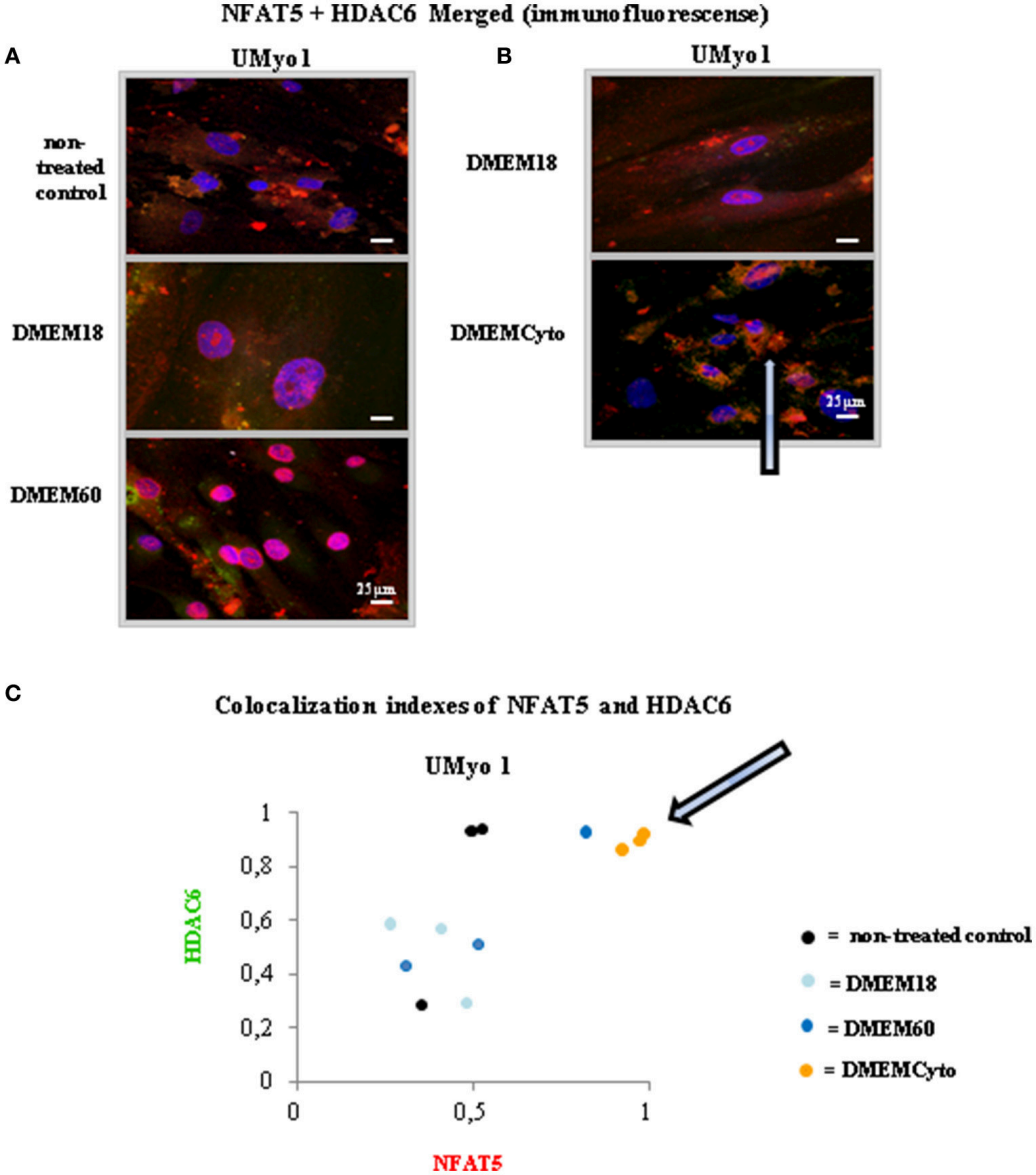
Study of NFAT5 colocalization with HDAC6 in unaffected myoblasts exposed to hyperosmolar or pro-inflammatory conditions. **(A,B)** Immunofluorescent staining was performed in UMyo1 (unaffected myoblasts) with NFAT5 Goat antibody and HDAC6 antibody, followed respectively by a Cy3 (red)-labeled secondary antibody and Alexa-488 (green)-labeled secondary antibody. Myonuclei were counterstained with DAPI staining (blue). NFAT5 with HDAC6 colocalization was only observed in UMyo1 exposed for 24 h to DMEMCyto. Colocalization of red and green dyes yielded a yellow to orange signal (white arrow). **(C)** Colocalization measurements were performed in UMyo1 by means of Volocity across all images. The graph represents colocalization indexes of NFAT5 and HDAC6 after exposure to DMEM18, DMEM60 and DMEMCyto for 24 h. NFAT5 with HDAC6 colocalization was only observed in UMyo1 exposed for 24 h to DMEMCyto (white arrow) (*n* = 3).

By confocal microscopy, NFAT5 colocalization with HDAC6 was studied in UMyo1 exposed to hyperosmolar or cytokine stress. Colocalization of red and green dyes yielded a yellow to orange signal. Colocalization was measured across the whole image by Volocity and depicted in a diagram. Results are displayed in Figure 4.

In UMyo1 exposed to DMEM18 or DMEM60, both red (NFAT5) and green (HDAC6) signals were distinctly present after superposition. Colocalization with HDAC6 was absent (Figures 4A,C). Exposure to DMEMCyto induced NFAT5 colocalization with HDAC6, visible as a yellowish coloration in the cytoplasm of the cells, after merging the red and green detection channels. Displaying the colocalization indexes of NFAT5 and HDAC6 in a graph after exposure to DMEM18, DMEM60 or DMEMCyto yielded the same results (arrows, Figures 4B,C).

### NFAT5 colocalizes with HDAC6 in PM and DM, but not in IBM

**Figure 5:**
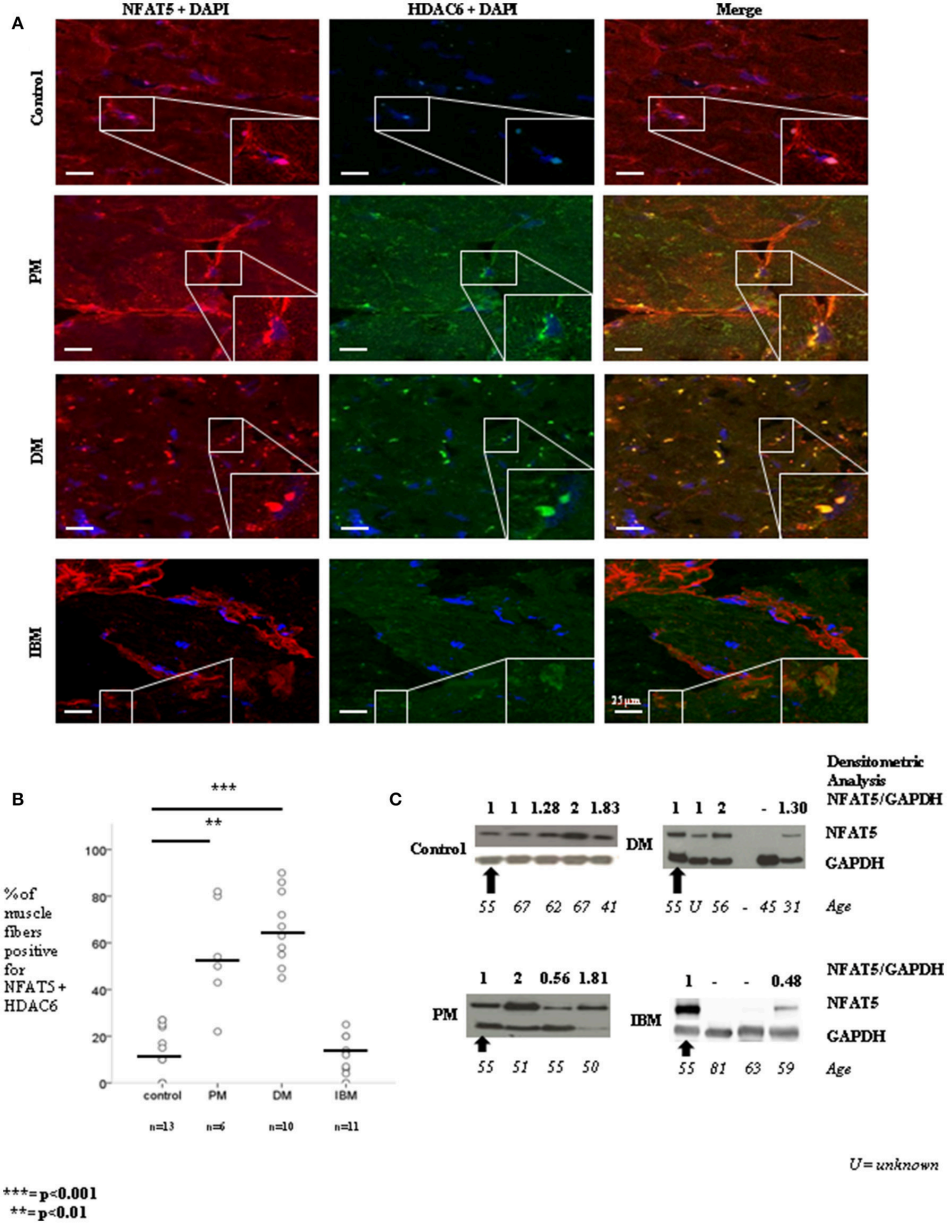
Study of NFAT5 localization and expression in control, PM, DM, and IBM biopsies **(A,B)** Immunofluorescent staining was performed in control, PM, DM, and IBM biopsies with NFAT5 Goat antibody and HDAC6 antibody, followed respectively by a Cy3 (red)-labeled secondary antibody and Alexa-488 (green)-labeled secondary antibody. Myonuclei were counterstained with DAPI staining (blue). In PM, and DM, NFAT5 colocalized with HDAC6, as displayed in the rectangular subsets. This is visible as yellow structures next to the nucleus. Absence of NFAT5 colocalization with HDAC6 was noticed in IBM. By quantitative analysis of confocal microscopy images, NFAT5 and HDAC6 colocalized in PM (*p* < 0.01) and DM (*p* < 0.001), but not in IBM. **(C)** By WB of total protein extraction of biopsies from control individuals and patients diagnosed with PM, DM, and IBM, different NFAT5 protein levels were measured. PM merely displayed NFAT5 protein levels in the same range as controls. In DM, 1/3 patients had no detectable NFAT5 protein levels. In IBM, 2/3 patients had no detectable levels of NFAT5 protein.

As visualized by IHC, 2/6 patients diagnosed with PM had a very discrete, diffuse NFAT5 surface staining. In DM, similar features were observed as in PM. In IBM, 4/8 patients expressed a positive muscle fiber surface NFAT5 staining. In IIM, NCAM (CD56) positive fibers (regenerating fibers) showed an absence of NFAT5 muscle fiber surface staining. The vast majority of inflammatory cells were negative for NFAT5 in IIM biopsies (Figure 5A and Supplementary Table 6).

By quantitative analysis of confocal microscopy images, NFAT5 and HDAC6 colocalized in PM (*p* < 0.01) and DM (*p* < 0.001), but not in IBM (Figures 5A,B). By WB of total lysates of patients' biopsies, NFAT5 was expressed in unaffected individuals free of any disease, where NFAT5 expression ranged from once to twice amount of NFAT5 protein. One individual was randomly selected as the standard individual (black arrow) and used in WB of PM, DM and IBM patients' biopsies, allowing for a comparison between blots. In PM, two out of three patients had a NFAT5 protein expression similar to controls. One patient had a decreased NFAT5 protein expression. In DM, NFAT5 protein expression was in the same range as controls and only one individual had no detectable levels of NFAT5 protein. In IBM, two out of three patients had no detectable levels of NFAT5 protein (Figure 5C).

**Figure 6:**
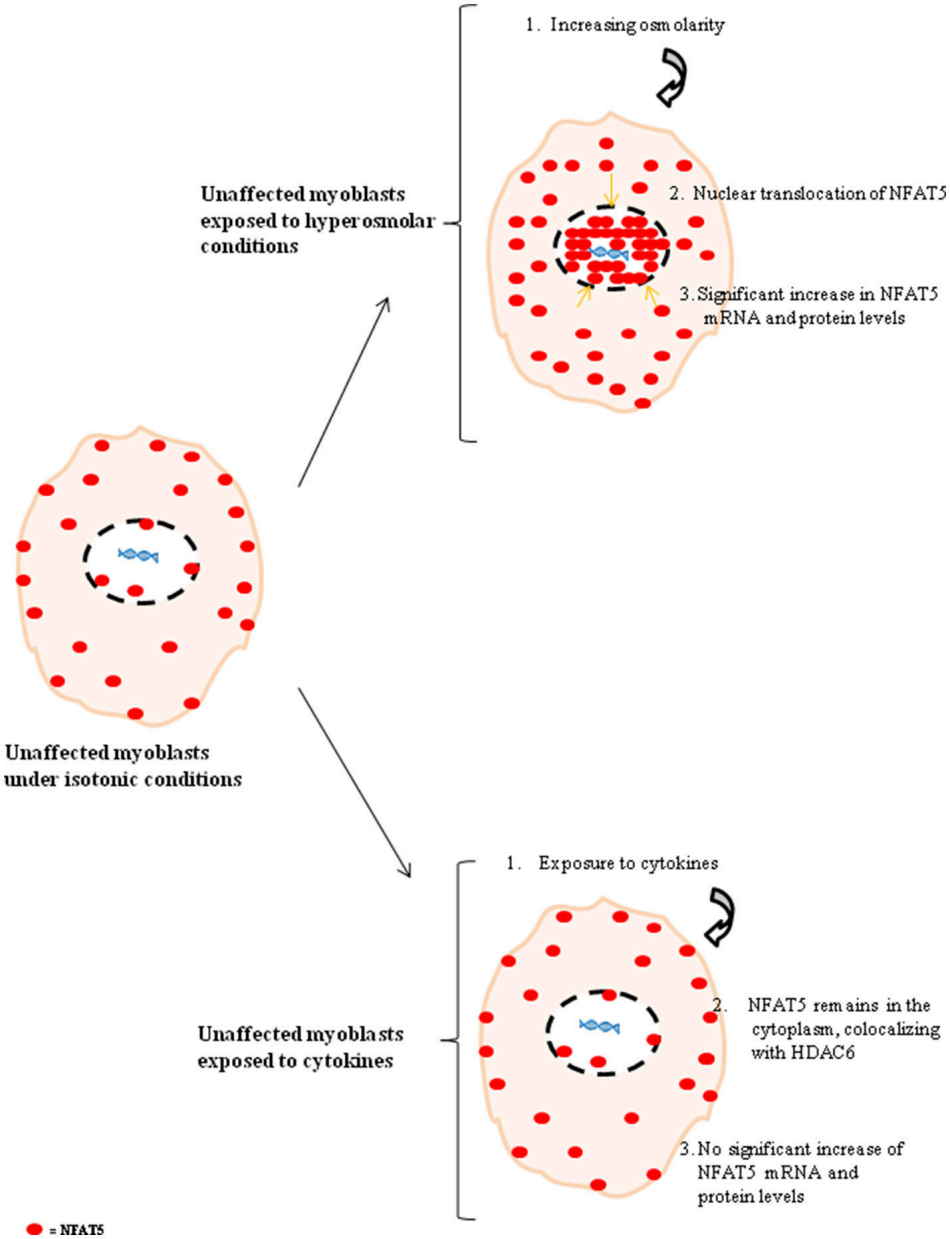
Schematic overview of NFAT5 localization and expression in unaffected myoblasts exposed to hyperosmolar or pro-inflammatory conditions. In unaffected myoblasts under isotonic conditions, NFAT5 is located in the cytoplasm were it is attached to the plasma membrane and resides in the cytoplasm. Increased osmolarity in these myoblasts induced NFAT5 translocation to the nucleus, with subsequent NFAT5 mRNA and protein level increase (upper myoblast). In unaffected myoblasts exposed to pro-inflammatory cytokines IFN-γ with IL-1β, NFAT5 was not translocating to the nucleus and NFAT5 mRNA and protein level were not increased (lower myoblast).

Figure 6 presents a schematic overview of the obtained results in NFAT5 physiology in unaffected myoblasts exposed to hyperosmolar or cytokine enriched DMEM solutions.

## Discussion

This study describes two main findings in NFAT5 physiology in unaffected muscle stem cells. Firstly, NFAT5 is translocated to the myonuclei and upregulated in myoblast cell cultures from unaffected individuals exposed to hyperosmolar DMEM solutions. Specifically in DMEM with an osmolarity increased by 60 mM, myoblasts display increased expression of NFAT5 mRNA and protein (IHC, WB, RT-qPCR). Secondly, after exposure of unaffected myoblasts to DMEM supplemented with cytokines IFN-γ with IL-1β, NFAT5 translocation to the myonuclei is not detectable. Instead, NFAT5 colocalizes with HDAC6 in the cytoplasm of myoblasts. NFAT5 mRNA and protein levels are not increased.

NFAT5 localization has been described in the past as being merely nuclear in myoblasts, after staining with NFAT5 Rabbit antibody for IHC (O'Connor et al., 2007). In the current study, NFAT5 is merely localized in the cytoplasm of myoblasts after staining with NFAT5 Goat for IHC. This observation may be explained by NFAT5 antibody specificity. NFAT5 Goat displays specific binding. NFAT5 staining is absent from both the cytoplasm and the nucleus after siRNA NFAT5 studies. NFAT5 Rabbit seems to display aspecific binding in the nucleus and to a lesser extend in the cytoplasm of myoblasts after siRNA NFAT5.

Our first observation corroborates with exposure of myoblasts to mannitol, a membrane-impermeant osmotic protein, where the NFAT5 reporter increases with increasing hyperosmolar mannitol concentrations (O'Connor et al., 2007). In the present study, adding hyperosmolar NaCl changes the cell environment to a hyperosmolar stressful setting. This leads to water drainage out of the cell by osmosis (Lang et al., 1998). To counteract this deleterious effect, NFAT5 is activated and translocates to the myonucleus where it binds with DNA (Ko et al., 2000; Dahl et al., 2001), with subsequent upregulation of NFAT5 mRNA and NFAT5 protein. NFAT5 is not colocalizing with HDAC6 in this study. This may point to a normal NFAT5 physiology in unaffected myoblasts after exposure to hyperosmolar conditions.

In the second observation, NFAT5 translocation could not be detected in unaffected myoblasts triggered by DMEM enriched with cytokines IFN-γ with IL-1β (IHC and WB). IFN-γ has been detected in muscle tissue of all the major subtypes of IIM (Figarella-Branger et al., 2003; Preuße et al., 2012). IFN-γ, IL-1β, and TNF-α were found to be markedly increased in IBM muscle compared to PM and DM tissue (Schmidt et al., 2008). Cytoplasmic NFAT5 was detected by IHC and WB, whereas NFAT5 was absent from the nucleus both in IHC and WB. By means of quantitative IHC, cytoplasmic NFAT5 colocalized with HDAC6. In NFAT5 physiology, entry into the nucleus depends of the nuclear localization system (NLS) localized in an amino acid cluster with residues 202 to 204. Mutations in this cluster abolish nuclear import of NFAT5 (Tong et al., 2006). NFAT5 amino acid modifications have been described for NFAT5, such as increased phosphorylation at threonine 135 (Gallazzini et al., 2011) or sumoylation after hyperosmolar stress (Kim J. A. et al., 2014). In the current study, NFAT5 may not be able to translocate to the nucleus after cytokine stimulation by alteration of the NLS or by conformational change of NFAT5. In unaffected myoblasts, this might be a mechanism to orchestrate the cell's appropriate response to hyperosmotic vs. pro-inflammatory conditions leading to a different downstream factor activation scheme (De Paepe et al., 2016). NF-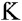
B is upregulated in myoblasts after exposure to pro-inflammatory cytokines. Under these conditions, it interferes with proteins involved in myogenesis (Langen et al., 2001). In this study, exposure of unaffected myoblasts to DMEM garnished with IFN-γ + IL-1β results in NFAT5 being localized in the cytoplasm and colocalizing with HDAC6, possibly pointing to NFAT5 interacting with HDAC6. HDAC6 is involved in cell motility and regulates heat shock protein 90 (Boyault et al., 2007; Zhang et al., 2007). Besides, HDAC6 is downregulated in embryonic stem cells during differentiation toward the myogenic cell lineage (Lee et al., 2015).

By WB of fractionated cell extracts in UMyo, three bands for NFAT5 are noticed, located around 170 kDa. In UMyo control cells, two bands are located in the cytoplasmic compartment and one band in the myonucleus. After hyperosmolar treatment, the two bands located in the cytoplasmic fraction disappear. The five following isotypes have been described for NFAT5: NFAT5a, NFAT5b, NFAT5c, NFAT5d1, and NFAT5d2 obtained by alternative splicing. Eisenhaber et al. (2011) describe diffusion to the nucleus of the plasma-membrane bound fraction of NFAT5a after exposure to salt stress of 350 mosm/L. This condition induces nuclear translocation of all isoforms following a linear curve with time, with complete migration to the nucleus of NFAT5b and NFAT5c (Eisenhaber et al., 2011). By consensus, isoform NFAT5c has been chosen as the canonical sequence and has a molecular weight of 166 kDa. Isoform NFAT5a weights 158 kDa (Germann et al., 2012). Isoform NFAT5b displays a molecular weight of 11 kDa (Germann et al., 2012). NFAT5 isoforms d1 and d2, also called NFAT5z1 and NFAT5z2 or NFAT5d and NFAT5e, have very similar molecular weights of respectively 167.7 kDa and 167.8 kDa (Xiao et al., 2015).

NFAT5 is a key regulator of both myoblast migration and differentiation during skeletal muscle myogenesis. Migratory defects in ^*NFAT*5+/−^ myoblasts are described alongside a decreased number of differentiated myoblasts during early differentiation in dominant-negative NFAT5 cells (O'Connor et al., 2007). Myofiber regeneration is a hallmark of DM and PM and regeneration is known to be impaired in IBM (Morosseti et al., 2006). Undetectable NFAT5 protein levels in almost all IBM patients is noticed in this current study, when studying NFAT5 expression by WB.

Combination of cytokine with hyperosmolar NaCl, possibly explaining the interplay between both conditions, led to complete cell death and was not pursued.

In summary, we describe normal NFAT5 localization and expression in unaffected myoblasts exposed to hyperosmolar stress with NFAT5 translocation to the nucleus, increased NFAT5 mRNA and protein levels. In unaffected myoblasts exposed to cytokine stress, NFAT5 localization and expression are impaired, with NFAT5 mainly located in the cytoplasm. In PM and DM patients' biopsies, NFAT5 remains within the normal range, whereas in IBM, an absence of NFAT5 expression can be seen. As NFAT5 is a core protein to myogenesis, our findings may help further our understanding of impaired muscle regeneration in myositis.

## Ethics statement

This study was carried out in accordance with the recommendations of Medical Ethics Committees at Ghent University Hospital and Antwerp University Hospital, as well as the RWTH Aachen Medical School Ethics Committee. Written informed consent was obtained from all subjects, in accordance with the Declaration of Helsinki. The protocol was approved by the Medical Ethics Committees at Ghent University Hospital and Antwerp University Hospital and the RWTH Aachen Medical School Ethics Committee.

## Author contributions

SH: Carried out the literature search and was responsible for the study design, data collection, data analysis, and interpretation, generating figures and writing the manuscript; ED, JV, OD and JD: Directed the study design, performed supervision, data analysis and interpretation, and assisted in the writing process; GV: Analyzed the data, while AG, BD, EN, LW, JW, JS both collected and analyzed the data. All authors contributed to the revision of the manuscript in terms of content.

### Conflict of interest statement

JS has received personal compensation for activities with Bayer, Biotest, CSL Behring, Novartis, and Octapharma as a consultant, both honoraria, and research grants. The other authors declare that the research was conducted in the absence of any commercial or financial relationships that could be construed as a potential conflict of interest.

## Abbreviations

BLAST: Basic local alignment search tool
DM: dermatomyositis
DMEMCyto: DMEM supplemented with pro-inflammatory cytokines IFN-γ + IL-1β
DMEM18: DMEM enriched with NaCl, increasing osmolarity by 18 mM
DMEM60: DMEM enriched with NaCl, increasing osmolarity by 60 mM
FITC: fluorescein isothiocyanate
GAPDH: glyceraldehyde 3-phosphate dehydrogenase
HDAC6: histone deacetylase 6
HSP70: heat shock protein 70
HSP90: heat shock protein 90
IBM: inclusion body myositis
IF: immunofluorescence microscopy
IFN-γ: interferon gamma
IIM: idiopathic inflammatory myopathies
IL-1β: interleukin 1 beta
LTβ: lymphotoxin beta
MIQE: minimum information for publication of quantitative real-time PCR experiments
NaCl: sodium chloride
NCAM: neural cell adhesion molecule
NFAT5: nuclear factor of activated T-cells 5
PARP: poly (ADP-ribose) polymerase
PM: polymyositis
RT-qPCR: quantitative real-time PCR
siRNA: silencing RNA
UMyo: unaffected myoblasts
WB: Western blotting.
