# Supplementary figures and images for: Localization and Expression of Nuclear Factor of Activated T-Cells 5 in Myoblasts Exposed to Pro-inflammatory Cytokines or Hyperosmolar Stress and in Biopsies from Myositis Patients
Source: Front Physiol. 2018 Feb 21;9:126. doi: 10.3389/fphys.2018.00126 (PMC5826317; doi:10.3389/fphys.2018.00126)

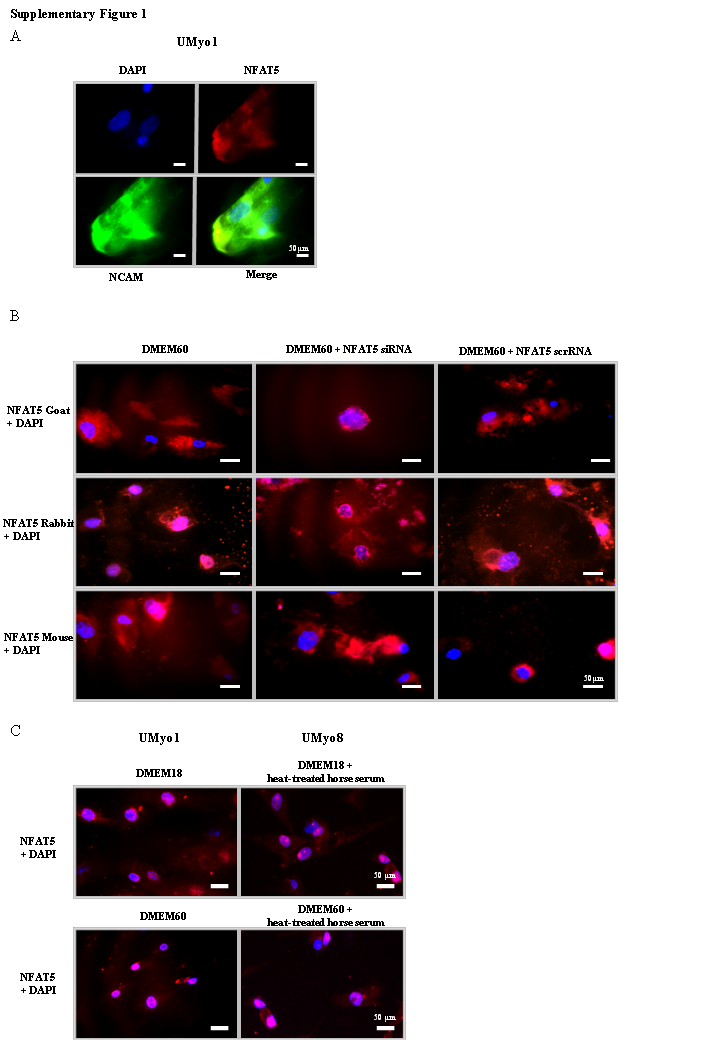

Supplement: Supplementary file 1 [file Image1.png]

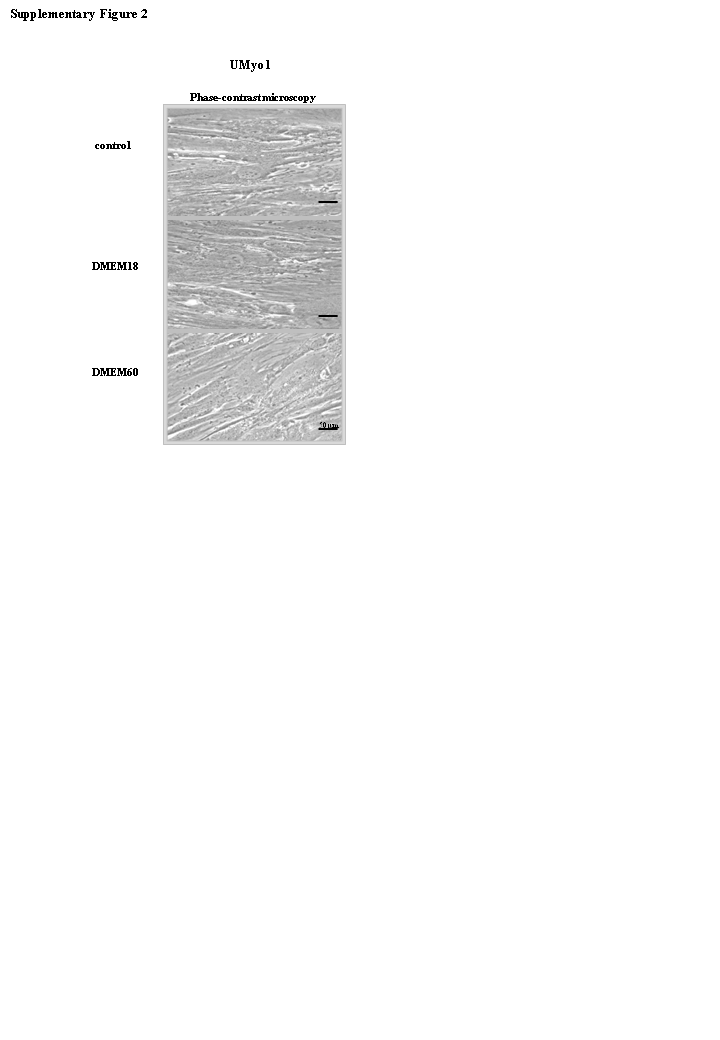

Supplement: Supplementary file 2 [file Image2.png]

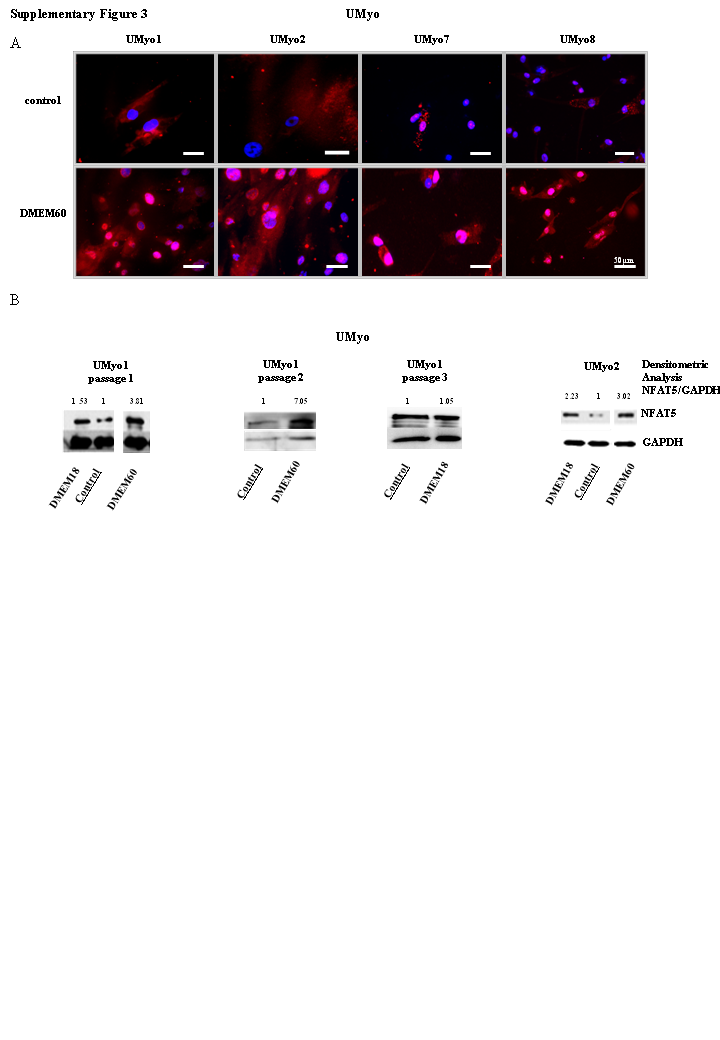

Supplement: Supplementary file 3 [file Image3.png]
