# Supplementary material for: Localization and Expression of Nuclear Factor of Activated T-Cells 5 in Myoblasts Exposed to Pro-inflammatory Cytokines or Hyperosmolar Stress and in Biopsies from Myositis Patients
Source: Front Physiol. 2018 Feb 21;9:126. doi: 10.3389/fphys.2018.00126 (PMC5826317; doi:10.3389/fphys.2018.00126)
Supplement: Supplementary file 4 [file Table1.pdf]

Localization and Expression of Nuclear Factor of Activated T-cells 5 in Myoblasts Exposed to Pro-inflammatory Cytokines or Hyperosmolar Stress and in Biopsies from Myositis Patients

Running title: NFAT5 in Myoblasts and Myositis

Sandrine Herbelet[1,*], Elly De Vlieghere[2], Amanda Gonçalves[3], Boel De Paepe[1], Karsten Schmidt[4], Eline Nys[1], Laurens Weynants[1], Joachim Weis[5], Gert Van Peer[6], Jo Vandesompele[6], Jens Schmidt[4], Olivier De Wever[2], Jan L. De Bleecker[1,*]

*** Correspondence:**

Corresponding authors: Sandrine.Herbelet@UGent.be and Jan.DeBleecker@UGent.be,
Tel: 00.32.9332.89.84 and Fax: 00.32.9332.49.71

Supplementary Table 1. Cell culture data

Not applicable (N/A).

Primary myoblast culture	Status	Provided by	Age (years)	Gender	Mutation/ Deletion
**UMyo 1**	Unaffected	Myobank (F)	16	Unknown	N/A
**UMyo 2**	Unaffected	Myobank (F)	16	Unknown	N/A
**UMyo 7**	Unaffected	Göttingen (D)	Unknown	Unknown	N/A
**UMyo 8**	Unaffected	Göttingen (D)	Unknown	Unknown	N/A

Supplementary Table 2. Primary antibodies used in immunohistochemistry

NFAT5= Nuclear Factor of Activated T-cells 5; NCAM= neural cell adhesion molecule; vWF= von Willebrand Factor; HDAC6= histone deacetylase 6. Antibodies directed to CD56 (NCAM) were applied to identify regenerating muscle fibers. CD3 stained T-cells, CD4+ and CD8+ provided a distinction between CD4+ cells and CD8+ cytotoxic T-cells. CD68 localized macrophages alongside with dendritic cells, vWF binds to endothelial cells and HDAC6 (histone deacetylase 6) is involved in cell motility.

Antigen	Primary antibody	Clone	Concentration	Source
**CD3 (all T cells)**	Mouse monoclonal IgG1	F7.2.38	1/100	Dako
**CD4**	Mouse monoclonal IgG1	MT310	2 µg/mL	Dako
**CD8**	Mouse monoclonal IgG1	C8/144B	9 µg/mL	Dako
**CD56 (=NCAM)**	Rabbit polyclonal	/	1 µg/mL	Merck
**CD68**	Mouse monoclonal IgG1	KP1	2,2 µg/mL	Abcam
**HDAC6**	Rabbit monoclonal IgG	D2E5	1/200	Cell Signaling
**NFAT5 (V-18)**	Goat polyclonal (IgG)	/	10 µg/mL	Santa-Cruz
**NFAT5 (F-9)**	Mouse monoclonal IgG2a	F-9	2µg/mL	Santa-Cruz
**NFAT5 SP5110P**	Rabbit polyclonal (IgG)	/	0,5µg/µL	Acris Antibodies
**vWF**	Mouse monoclonal IgG1	F8/86	2,3µg/mL	Dako

Supplementary Table 3. Patient data

Polymyositis (PM); dermatomyositis (DM); inclusion body myositis (IBM); non-necrotic invaded fiber (NNIF).

Patient	Muscle pathology	Age (years)	Gender	Muscle damage	Inflammation	Concomitant disease
**Patient 1**	PM (NNIF +)	51	Female	Severe	Severe	Graves, SLE
**Patient 2**	PM (NNIF +)	50	Female	Mild	Mild	Diabetes
**Patient 3**	PM (NNIF +)	38	Male	Weak	Weak	None
**Patient 4**	PM (NNIF +)	37	Female	Mild	Mild	None

**Patient 5**	PM (NNIF +)	73	Female	Severe	Severe	Diabetes,Hypo-thyroidism
**Patient 6**	PM (NNIF +)	55	Male	Mild	Mild	Rheumatoid arthritis
**Patient 7**	DM	66	Male	Mild	Mild	Unknown
**Patient 8**	DM	65	Female	Severe	Severe	Unknown
**Patient 9**	DM	45	Male	Severe	Severe	Unknown
**Patient 10**	DM	42	Female	Severe	Weak	Unknown
**Patient 11**	DM	57	Male	Weak	Weak	Unknown
**Patient 12**	DM	49	Female	Unknown	Unknown	None
**Patient 13**	DM	73	Male	Unknown	Unknown	None
**Patient 14**	DM	56	Male	Unknown	Unknown	Unknown
**Patient 15**	DM	31	Female	Unknown	Unknown	Unknown
**Patient 16**	IBM	70	Female	Severe	Severe	None
**Patient 17**	IBM	63	Male	Mild	Weak	None
**Patient 18**	IBM	73	Male	Severe	Severe	None
**Patient 19**	IBM	59	Male	Severe	Severe	Diabetes+ACE inhibitors
**Patient 20**	IBM	70	Male	Severe	Severe	None
**Patient 20**	IBM	68	Male	Severe	Severe	None
**Patient 22**	IBM	71	Male	Severe	Severe	Arterial hypertension
**Patient 23**	IBM	81	Female	Severe	Severe	None
**Patient 24**	IBM	59	Female	Severe	Severe	None

**Patient 25**	IBM	57	Female	Severe	Severe	None
**Patient 26**	IBM	62	Female	Severe	Severe	Neuropathy

Supplementary Table 4. Primary antibodies used in Western-blotting

GAPDH= glyceraldehyde 3-phosphate dehydrogenase, PARP= poly (ADP-ribose) polymerase

Antigen	Primary antibody	Clone	Concentration	Source
**GAPDH**	Mouse monoclonal IgM	71.1	0.4µg/mL	Sigma- Aldrich
**Histone H3**	Mouse monoclonal IgG3	24834	5µg/mL	Cell Signaling
**NFAT5 (F-9)**	Mouse monoclonal IgG2a	F-9	2µg/mL	Santa-Cruz
**PARP**	Mouse monoclonal IgG1	4C10-5	0.5µg/mL	BD Biosciences

Supplementary Table 5. Genes used in RT-qPCR

UBC= ubiquitin C; *HPRT1*= hypoxanthine phosphoribosyltransferase 1; *B2M*= beta-2 microglobulin; *RPL13A*= 60S ribosomal protein L13a; *YWHAZ*= Tyrosine 3-Mono-oxygenase/Tryptophan 5-Mono-oxygenase Activation Protein, Zeta; *SDHA*= Succinate dehydrogenase complex, subunit A, *HMBS*= hydroxymethylbilane synthase, *TBP*= TATA-binding protein, *AluSq*= Alu restriction enzym, *AluSx1*= interspersed repeat subfamily.

Gene	Primer	Concentration	Source
NFAT5	PrimePCR SYBRGreen Assay qHsaCID0015734 intron-spanning	1x	Bio-Rad
UBC	F:ATTTGGGTCGCGGTTCTTG	1,25pmol	IDT
	R:TGCCTTGACATTCTCGATGGT	1,25pmol	IDT
HPRT1	F: TGACACTGGCAAAACAATGCA	1,25pmol	IDT
	R: GGTCCTTTTCACCAGCAAGCT	1,25pmol	IDT
B2M	F: TGCTGTCTCCATGTTTGATGTATCT	1,25pmol	IDT
	R: TCTCTGCTCCCCACCTCTAAGT	1,25pmol	IDT
RPL13A	F: CCTGGAGGAGAAGAGGAAAGAGA	1,25pmol	IDT
	R: CCTGGAGGAGAAGAGGAAAGAGA	1,25pmol	IDT
YWHAZ	F: ACTTTTGGTACATTGTGGCTTCAA	1,25pmol	IDT
	R: CCGCCAGGACAAACCAGTAT	1,25pmol	IDT
SDHA	F: TGGGAACAAGAGGGCATCTG	1,25pmol	IDT
	R: CCACCACTGCATCAAATTCATG	1,25pmol	IDT
HMBS	F: GGCAATGCGGCTGCAA	1,25pmol	IDT
	R: GGGTACCCACGCGAATCAC	1,25pmol	IDT
TBP	Unknown	1,25pmol	[49]
AluSq	Unknown	1,25pmol	[49]
AluSx1	Unknown	1,25pmol	[49]

Supplementary Table 6. NFAT5 immunofluorescent staining in patients' muscle biopsies

Dermatomyositis (DM); inclusion body myositis (IBM); polymyositis (PM); scoring: - = absent, + = weak positive, ++ = mild positive, +++ = strongly positive, ( ) = minority of cells, N/A = not applicable/not present.

Staining	Control	DM	IBM	PM
**Myonuclei/Perinuclear**	+	+++	+	++
**Sarcolemma**	-/(+)	- /(+)	+/-	-/+
**CD3+ T-cells**	-	-	+/-	-
**CD4+ T-cells**	-	-/(+++)	+/-	-/+
**CD8+ T-cells**	-	-	-	-
**CD68 + cells**	N/A	-/+	-/+	N/A
**NCAM+ muscle fibers**	N/A	-	-/+	-/(+)
**Endothelial cells**	-	-/+	-/(+)	-/(+)

Supplementary Figure 1 Additional tests

Immunofluorescence was performed in UMyo (unaffected myoblasts) with NFAT5 Goat antibody and NCAM antibody, followed respectively by a Cy3 (red)- labelled secondary antibody and Alexa-488 (green)- labelled secondary antibody. Myonulei were counterstained with DAPI staining (blue). **(A):** In UMyo 1 control myoblasts, NCAM and NFAT5 are expressed in almost all cells. (n=3). **(B):** Myoblasts were exposed to DMEM60 (DMEM with a total increase in osmolarity of 60mM), DMEM60 + NFAT5 siRNA and to DMEM60 + NFAT5 scrRNA, assessing NFAT5 antibody specificity. NFAT5 Rabbit shows aspecific binding in the myonuclei. NFAT5 Mouse binds aspecifically in the cytoplasm. NFAT5 Goat is the most suited antibody for IHC, albeit some minor punctate signals remain at long exposure time (n=1). **(C):** NFAT5 expression is not influenced by addition of heat-treated horse serum in UMyo8 after addition of DMEM18 or DMEM60 (n=3).

Supplementary Figure 2 Confocal microscopy

By confocal microscopy, untreated UMyo1 myoblasts have a spindle-shaped morphology (n=3).

Supplementary Figure 3 Variation in NFAT5 localization and expression amongst unaffected cell cultures

(A): Immunofluorescence was performed in UMyo (unaffected myoblasts) with NFAT5 Goat antibody, followed by a Cy3 (red)-labelled secondary antibody. Myonulei were counterstained with DAPI staining (blue). In all UMyo, NFAT5 nuclear staining is induced after adding DMEM60 (DMEM with a total increase in osmolarity of 60mM) (n=1). **(B):** By WB, an increase in NFAT5 protein levels is observed in all tested UMyo (n=3).
